# Impact of educational intervention on willingness-to-pay for health insurance: A study of informal sector workers in urban Bangladesh

**DOI:** 10.1186/2191-1991-3-12

**Published:** 2013-04-29

**Authors:** Jahangir AM Khan, Sayem Ahmed

**Affiliations:** 1 Health Economics and Financing Research Group, International Centre for Diarrhoeal Disease Research, Bangladesh (icddr,b), Dhaka 1212, Bangladesh; 2 Centre of Excellence for Universal health Coverage, icddr,b and James P Grant School of Public health, Dhaka 1212, Bangladesh; 3 Department of Learning, Informatics, Management and Ethics, Karolinska Institute, Berzelius va¨g 3, 171 77, Stockholm, Sweden

**Keywords:** Healthcare financing, Health insurance, Education, Willingness to pay, Impact

## Abstract

**Background:**

The reliance on out-of-pocket payments for health services leads to a catastrophic burden for many households in Bangladesh. The World Health Organization suggests that risk-pooling mechanisms should be used for financing healthcare. Like many low-income countries (LIC), a large share of employment in Bangladesh is in the informal sector (88%). Inclusion of these workers in health insurance is a big challenge. Among other barriers, the “literacy gap” for health insurance” is a reason for the low insurance uptake in Bangladesh. The aim of this study is, therefore, to assess the impact of an educational intervention on willingness-to-pay (WTP) for health insurance among informal sector workers in urban Bangladesh.

**Method:**

An educational intervention on occupational solidarity and health insurance is offered to groups of informal workers. Educational sessions take place once a week (3–4 hours) during three subsequent weeks for each occupational group. For assessing the impact of the educational intervention, WTP for joining health insurance using occupational solidarity between workers in “pre- and post-treatment” periods as well as between “control and treatment” groups were compared. Multiple-regression analysis is applied for predicting WTP by educational intervention, while controlling for demographic and socioeconomic characteristics.

**Results:**

The coefficient of variation (CoV) of the WTP is estimated in control and treatment groups and expected to be lower in the latter. The WTP for health insurance is higher (33.8%) among workers who joined the educational intervention in comparison with those who did not (control group). CoV of WTP is found to be generally lower in post-treatment period and in treatment group compared to pre-treatment period and control group respectively.

**Conclusion:**

Educational interventions can be used for increasing demand for health insurance scheme using occupational solidarity among informal sector workers.

## Background

The poor in Bangladesh face many barriers to access health care. Private health expenditure constitutes 64.3% of total healthcare expenditure of which 97.4% is covered through out of pocket payments (WHO, 2011). Reliance on out-of-pocket payment for health services likely leads to a catastrophic burden for many households in Bangladesh and other Asian countries [1,2]. Pre-payment mechanisms of healthcare financing, like health insurance, is thus important for health-related financial protection in the population, particularly for those in vulnerable situations.

According to World Health Organization, risk-pooling mechanisms should be used for financing healthcare to achieve universal coverage n [3]. In health financing policy, society risk-pooling mechanism appears to be ignored in policy prescriptions for many low income countries [3,4]. The inclusion of poor and informal sector workers in these mechanisms appears to be a challenge that has yet to be addressed [5,6]. Community-based health insurance has been recommended for informal sector workers for ensuring their healthcare [7]. Occupation has been considered as a unit of solidarity in many social movements and historically such solidarity has been a basis of health insurance development [8]. According to Oxford Learner’s dictionary, Solidarity (with somebody) means support by one person or group of people for another because they share feelings, opinions, aims, etc. In the current context, the workers in same occupation (like, rickshaw-pullers) or same sector (like, informal sector) are expected to share common feelings for financial risk protection for health.

Of total employments in Bangladesh, 88% takes place in informal sector, of which, 48% is in the non-agricultural sector. The informal sector alone contributes to 63.6% of total GDP and 75.3% of this comes from the non-agricultural sector. This means that the agricultural sector dominates in terms of the number of workers and non-agricultural sector dominates in terms of income [9]. Considering the size of informal sector labor force and the contribution to the economy and challenges of healthcare financing, an effort to bring health insurance to informal laborers should be initiated.

A literature review by the International Labor Organization and Micro Insurance Initiation facility identified a number of barriers that restrict potential clients from joining health insurance schemes in low- and middle-income countries. Among the barriers, the “literacy gap” or the lack of knowledge about insurance (mechanism, utility etc.) was found to be important [10-12]. In Bangladesh, studies on knowledge about health insurance are not readily found; however, there are indications that a literacy gap for health insurance exists. The prevalence of health insurance coverage among the general population, either rich or poor, is very low. The National Health Accounts of Bangladesh shows that only 0.10% of healthcare expenditure is borne by pre-payments mechanism [13].

Micro Health Insurance (MHI) is an emerging sector which is strongly linked to the microcredit movement in Bangladesh [14]. Currently, health insurance is offered by a number of micro-credit institutes like: Grameen, Kalyan and Sajida Foundation. In these programs, the potential clients purchase micro-health insurance if they borrow from the institutions. It implies that this micro-health insurance is not purchased by the clients on the basis of demand. Other organizations, like Gono-swasthyo Kendro (GK) offers health cards against which a benefit package is available to the card purchasers. Such an arrangement may reduce the financial risks of illness for the clients, but may not be insufficient for generating revenue for financing healthcare in a sustainable way. Organizations which currently offer micro health insurance inform the clients about the benefit package. Since these institutions function as third-party insurers and solely have the responsibility for management, informing clients about the mechanism of health insurance (like, risk pooling) and the importance of solidarity for combating healthcare expenditure through health insurance (like, social and community-based health insurance) may not be their primary concern. Research from Kenya has shown that the spirit of solidarity and the rationale for health insurance are some of the key issues for demand for health insurance in the informal sector [15]. While many health awareness (educational) programs for disease prevention and health promotion are available in Bangladesh, education about protection against financial risk during illness is not generally found. In such a condition, a comprehensive education intervention on health insurance, its mechanisms, utility and role of solidarity for informal sector workers of Bangladesh can be useful. The aim of this study is to assess the impact of an educational intervention on willingness-to-pay for health insurance using occupational solidarity among informal sector workers in urban Bangladesh.

## Methods

We have used a quasi experimental study design to assess the impact of an educational intervention on the willingness-to-pay (WTP) for joining health insurance. Specifically, we employ occupational cooperatives between workers in pre- and post-treatment periods as well as between control and treatment groups have been compared. Multiple regression analysis is applied for predicting WTP for the intervention.

### Population, sample and data

Informal workers, working or living in the areas around any insurance provider (Gonoswyastho Kendro) or any healthcare facility are considered to be the population of this study. Three occupational groups (rickshaw-puller, shop-keepers and restaurant workers) were selected as study participants in three locations in Dhaka (metropolitan city), Chandpur (district town) and Nobinagar/Savar (sub-district). These occupational groups have been selected for investigation considering that: i) the occupations are generally found in all urban areas in Bangladesh, ii) the practical situation such as the consent of the occupational group representative and the working environment allows for operating the educational intervention and iii) a control group can be identified in a practical context. The locations were selected from three levels of administrative hierarchy of Bangladesh for a national representation.

In the selected locations, we listed out the cooperatives and potential participants by transect walk and informal group discussion with the community members and leaders. Detailed information about the cooperatives (formal or informal) like location and address, representatives of the cooperatives and their contact number were collected during the listing procedure. Shops, restaurants and cooperatives of the rickshaw-pullers in the selected areas have been listed. A list of workers was provided by the representatives/leaders of the cooperatives (formal or informal). Considering the practical working environment of occupational groups in the intervention sites, assistance of occupational/community leaders was essential for getting workers as participants in the study. A number of inclusion and exclusion criteria were considered as well. The inclusion criteria comprised age (18 years or above) and experience (working in the same occupational at least last one year). If the worker was exposed by any health insurance or any health insurance education, he/she is excluded from the study.

The treatment and control groups were separated by a road or river so that the participants in the control group cannot be exposed by intervention through the participants of the treatment group. It needs to be emphasized here that during the listing procedure, we discussed with the community leaders about possible dissimilarities between the workers across the road or river and no potential difference was reported. For keeping similarities in socioeconomic characteristics between treatment and control groups, we considered only market places to be included in the sample from all occupational groups. Consequently, we received two separate lists of potential participants for control and treatment groups in each location. We randomly selected the participants in control and treatment groups from respective lists. In each area, it took 2 months for completing data collection including pre- and post-treatment periods. Post-treatment data was collected one month after the educational intervention has been completed for avoiding the immediate impact of intervention on participants [16,17]. In control groups, we collect data for one time. Data in all study areas has been collected during 15^th^ December 2010 – 15^th^ April 2011.

Our interest was to estimate mean values of WTP from three occupational groups in the three areas. The Central Limit Theorem suggests that at least 30 cases are required for calculating mean value with an assumption of normal distribution [18]. This implies that 30 workers in each of three occupations in three areas constitute 270 samples in each of control and treatment group, which means that at least a total of 540 samples are required for the study.

We invited all the participants (282) in the pre-treatment group to attend a three-day educational intervention. 25 of them were unable to attend the intervention program due to personal reasons. We also lost some participants (32) during the time of the educational intervention. The participants (8 in Dhaka, 9 in Chandpur and 15 in Nobinagar/Savar) who missed one or more days in three study areas were excluded as not properly exposed by the intervention. These participants (total 32 or 12.5%) are omitted from both pre- and post-treatment group in the analyses. However, in the control group, we have 100% response rate.

### Educational intervention procedure

Educational interventions on occupational solidarity and health insurance were offered. Educational sessions take place once a week (3–4 hours) during three subsequent weeks for each occupational group. The interventions used power point presentation (mostly pictorial), group session and general discussion. In the first day, it contains discussion about health condition, healthcare expenditure and current healthcare facilities of workers. In the second day, health insurance mechanisms and utility of health insurance have been discussed. Potential uses of occupational solidarity for developing health insurance scheme have been discussed in the third day. In the (Table 1), the intervention procedure is presented.

**Table 1:** Components of educational intervention

**Day**	**Topic**	**Aim**	**How**	**Lead by**
First	Importance of urban informal sector workers in Bangladesh	To make the participants understand the potential of contribution to economy and collectively meeting challenges of health	Power point presentation and discussion	The main facilitator
	Case study on Golam Kibria, an informal sector worker who got sick and its consequence on health, economy and family	To make understand the importance of good health on economy and family	Group discussion	Group moderator
	Current healthcare facilities of workers and its quality of service	To understand the current situation of healthcare access and quality of care for the workers under intervention and their level of satisfaction	Group discussion	Group moderator
Second	Current mechanism of healthcare financing, healthcare triangle, concept and utility of health insurance	To put into context of sustainable and self-dependent healthcare financing	Power point presentation and discussion	Main facilitator
	Insurance game	To make understand the risk-sharing mechanism	Game	Group moderator
	Roll-play	To distinguish the service and payment difference between non-insured and insured patients	Short drama	Jointly by educators
	Types of health insurance and its merits and demerits	To make understand the merits and demerits of different types of insurance (private for profit, NGO and community based)	Discussion	Group moderator
Third	History of social health insurance and recent development in low and middle income countries	To put the participants into global context and finding the position of Bangladesh	Power point presentation with discussion	Main facilitator
	Occupational cooperatives and/occupational solidarity	To understand the possibilities and challenges of using occupational cooperative/ solidarity for developing health insurance	Group discussion	Group moderator
	Open discussion (questions and answers)	To understand if the sessions could successfully meet the goals and to clarify any issues to the workers	Discussion	Main facilitators and all moderators

### Impact assessment

The impact of the educational intervention is assessed as the change (between pre- and post-treatment) and difference (between treatment and control) in the WTP measurement. Further, multiple regression analysis is applied for predicting WTP by educational intervention. WTP data has been collected in pre-treatment period for both control and treatment groups and also in post-treatment period in treatment group. WTP are captured using pre-fixed survey questionnaire.

Health insurance is a rare way of financing health scheme in Bangladesh. The latest Bangladesh National Health Accounts mentioned that only 0.1% of healthcare financing is connected to health insurance [13]. Further, the findings from informal discussion with the workers in control and treatment group show that none of them were exposed to any insurance education earlier. Our follow up with the workers in control group shows that they were not exposed to health insurance education or any information in this regard from any other sources during the study period. We, therefore, compared the post-treatment group with the control group once.

#### Willingness to pay

Using Contingent Valuation Method (CVM), WTP for health insurance is measured. This method has been earlier used in many studies [19-21].

CVM questions can be either open-ended or discrete [22]. In an open-ended valuation the respondents are asked to state their maximum WTP for the benefit and the technique most used is the so called “bidding game”. A bidding game resembles an auction, where a first bid is made to the respondent who either accepts or rejects. Depending on the answer, the bid is then lowered or increased until the respondent’s maximum WTP is reached. This bidding game approach is applied in the study for estimating the WTP for health insurance. In the recent years, “bidding game” has been employed in several studies for estimating WTP for health insurance in low- and middle income countries [20,23]. However, bidding game may be accompanied with estimation bias, which is a form of framing effect where the respondents’ answers are influenced by the first numbers presented in the bidding game [24]. On the contrary, there are studies which used bidding game, but observed no starting point bias [25,26].

For capturing the starting bids, we interviewed a number of workers from each occupational group. We found a range between 10 and 30 Bangladeshi Taka (BDT) which were put randomly in the questionnaire as the starting bids. The interviewer described the benefit package to the respondent and asked if he/she is willing to pay the amount that has been randomly placed as starting bid for the respective respondent. If the respondent agreed to pay the amount, then the interviewer raised the bid with higher amount. The interviewer continues until the respondent disagrees to pay higher amount. On the contrary, if the respondent disagrees to pay the amount of starting bid the interviewer lowered the amount and asked again about the willingness to pay of the respondent. This process continues until the respondent agreed to pay an amount including no payment. The benefit package, which is as same as that offered by Gonoshasthaya Kendra against a pre-paid membership card, was tested for investigating the WTP of workers for obtaining that package through a hypothetical health insurance. The product is described in the (Table 2).

**Table 2:** The service package of a real health insurance product

**Eligibility**	**Anyone on paying premium**
**Group or individual**	Family up to 4 members
**Period of services**	One year
**Outpatient**	Low income/poor
*Medical officer visit*	Free of cost
*Specialist visit*	60 BDT
**Inpatient**	
*Bed-Payment per day*	50 BDT
**Diagnostic tests**	
Ultrasonography	75-150 BDT
ECG	50 BDT
Most of the tests	Free of cost
Some tests	10 - 200 BDT
Blood transfusion of neonatal	500 BDT
Other treatment of neonatal	Free of cost
Normal delivery	100 - 500 BDT
Caesarean and other surgery	2000 - 3000 BDT
Orthopedic surgery	3000 - 4000 BDT
Appendicitis	100 BDT
Gall bladder operation	3000 BDT
**Medicine**	50% discount of MRP set by government

Finally, in the descriptive statistics, the knowledge level and attitude are captured by observing the frequency of response category to each corresponding question. The frequencies are then compared between “pre- and post-treatment” and “treatment and control” groups. Mean and coefficient-of-variation (CoV) of WTP for an insurance package are observed between the comparison groups. We expect WTP improve and CoV lower among workers who attend the educational intervention. The Cohen’s *d*, effect-size (ES) measurement is used for representing changes between pre-treatment and post-treatment and between control and post-treatment. Cohen (1965) showed that one useful option for measuring the effect size between dummy-coded groups or conditions (e.g., coded 1 for experimental and 0 for control) and scores on a continuous variable. The Cohen’s *d*, effect-size concentrate on the standardized difference between the sample means (*M*1 and *M*2) [27,28]. Cohen’s *d is* defined as,

$$d=\frac{M_{1}-M_{2}}{\delta_{\mathrm{Pooled}}}$$

Where, *δ*_*Pooled*_ is a pooled estimate of the population standard deviation. The ES is regarded as large when d = 0.5 and above, moderate when d = 0.2 to 0.5 and small when d= 0.2 and below [27,28].

#### Econometric model

In the regression model, we predict natural logged WTP by participation in intervention (main variable of interest) while controlling for demographic and socioeconomic characteristics. Insurance literature demonstrates that demand for health insurance is influenced not only by knowledge but also by other factors. Folland et al. (2007), in a theoretical model mentioned that premium, income or wealth, health status and risk of losing income are factors that can affect the demand for health insurance [29]. Similar factors have been indicated by researchers from their empirical investigations [30-33]. While assessing the impact of educational intervention, these other factors should be controlled. In the regression model, we therefore control for a range of variables, namely, demographic characteristics, institutional education level, and household income, experience of illness among household members, occupation and place of residence. The model below is tested in the analysis.

$$y=\alpha +\beta_{1}x_{1}+\beta_{2}x_{2}+\ldots \ldots \ldots \ldots +\beta_{n}x_{n}+\epsilon$$

Where, *y* denotes natural logged WTP for joining an insurance scheme, α is a constant, *x*_*1*_ indicates if the worker went through educational intervention with values 0 or 1 (0 = did not have educational intervention/control, 1 = had educational intervention/treatment), β_1_ is the coefficient that shows the magnitude and direction of relationship with *y*. *x*_*2*_ … …*x*_*n*_ denote the control variables. *β*_*2*_… …*β*_*n*_ denote adjacent coefficients to the corresponding variables and ϵ means error term. The model is tested for sensitivity by including and excluding any variables and by estimating the robust standard error. A series of diagnostic tests, like presence of heteroscedasticity, multicollinearity, omitted variables are carried out.

## Results

A description of characteristics of control and treatment groups is presented, followed by the impact assessment results. Impact assessment is described by presenting the WTP for insurance in treatment and control groups and the outcomes of econometric analyses.

### Characteristics of control and treatment groups

Demographic characteristics (age, gender, marital status and household size), institutional education level, economic condition (household income), health status (illness) and health expenditure are observed in control and treatment groups. The characteristics are presented in the (Table 3). Independent sample t-test of mean and proportion difference has been carried out for observing if there is any significant difference between control and treatment groups. Mean age of all workers in control group is 30.3 years and in treatment group is 30.8 years. No significant difference (p-value = 0.606) was observed between these two groups. Other characteristics also showed similarities between these two groups.

**Table 3:** Characteristics of sample in control and treatment groups

**Variables**	**Measurement**	**Rickshaw-puller**		**Shop-keeper**		**Restaurant worker**		**Total**	
		**Control**	**Treatment**	**Control**	**Treatment**	**Control**	**Treatment**	**Control**	**Treatment**
Age	Mean (SD^a)^) in years	32.1 (9.8)	34.65 (10.0)	26.9 (9.5)	28.0 (8.29)	32.0 (11.5)	30.2 (10.3)	30.3 (10.5)	30.78 (9.9)
	Sig. of mean difference	0.112		0.397		0.299		0.606	
Gender	Male (%)	100%	100%	96.8%	100%	90.8%	81.94%	95.9%	94.1%
	Sig. of proportion diff.	-		0.102		0.101		0.347	
Marital status	Married (%)	85.9%	79.4%	39.4%	34.1%	71.3%	58.3%	65.2%	55.9%
	Sig. of proportion diff.	0.281		0.475		0.088		0.034	
Household size	Mean (SD)	3.1 (0.98)	3.4 (1.0)	4.1 (1.85)	3.7 (1.35)	3.4 (1.4)	3.6 (1.5)	3.5 (1.5)	3.6 (1.3)
	Sig. of mean difference	0.299		0.239		0.477		0.849	
Institutional educational level	Less than one year (%)	75.0%	66.1%	11.7%	9.8%	48.3%	38.9%	44.7%	36.5%
	Sig. of proportion diff.	0.222		0.678		0.235		0.065	
	Up to primary level (%)	17.4%	30.9%	37.2%	28.0%	36.8%	34.7%	30.4%	31.0%
	Sig. of proportion diff.	0.045		0.196		0.788		0.871	
	More than primary level	7.6%	2.9%	51.0%	62.2%	14.9%	26.4%	24.9%	32.4%
	Sig. of proportion diff.	0.205		0.138		0.073		0.065	
Household income per equivalent adult^b)^	Mean (SD) in BDT^c)^	3151 (1376)	3129 (1436)	3360 (1562)	3735 (1740)	2447 (1112)	2975 (1754)	3024 (1419)	3308 (1675)
	Sig. of mean difference	0.94		0.227		0.084		0.103	
Health expenditure in last 6 months	Mean (SD) in BDT	1314 (49)	1814 (4944)	1838 (3893)	2176 (3893)	2066 (7059)	2041 (7344)	1734 (5399)	2021 (6159)
	Sig. of mean difference	0.527		0.655		0.983		0.581	
Illness in household	Yes (%)	89.1%	86.8%	82.9%	85.4%	89.7%	87.5%	87.2%	86.5%
	Sig. of proportion diff.	0.648		0.666		0.669		0.820	
Location	Metropolitan city (%)	32.6%	36.8%	34.0%	34.1%	33.3%	36.1%	33.3%	35.6%
	Sig. of proportion diff.	0.584		0.988		0.714		0.600	
	District (%)	34.8%	29.4%	34.0%	34.1%	35.6%	27.8%	34.8%	30.6%
	Sig. of proportion diff.	0.473		0.988		0.291		0.326	
	Sub-district (%)	32.6%	33.8%	31.9%	31.7%	31.0%	36.1%	31.9%	33.8%
	Sig. of proportion diff.	0.872		0.977		0.499		0.651	
Observations		92	68	94	82	87	72	273	222

Note:^a)^ SD means standard deviation, ^b)^ First adult, other adults and children are weighted as 1, 0.7 and 0.5 respectively (Source: OECD, 1982), ^c)^ BDT = Bangladeshi Taka.

### Impact assessment

Quantitative analyses include both descriptive statistics and statistical inference tests. Changes (pre- and post-treatment) and differences (control and treatment) in WTP for participating in such schemes are shown. Further, an output of econometric analysis where WTP (natural logged) has been predicted by participation in educational intervention (treatment) is presented.

#### Willingness to pay

Between pre- and post-treatment periods, mean WTP has increased in workers after intervention in all occupational groups. Paired t-test between pre- and post-treatment showed that the changes in average WTP were statistically significant (1% risk-level) in shop-keepers and boarder line significant in rickshaw-pullers (10% risk-level) (Table 4).

**Table 4:** Change in willingness-to-pay (mean and CoV) between pre- and post-treatment periods and within group effect sizes (Cohen’s d)

**Occupational group**	**Measurement**	**Mean**	**Sig. of mean difference (p-value)**	**ES (Cohen’s d)**	**Coefficient of variation**
Rickshaw-puller	Pre	23.8	0.109	−0.281	50.7
	Post	27.2			45.2
Shop-keeper	Pre	14.2	0.002	−0.501	92.4
	Post	20.3			57.1
Restaurant workers	Pre	17.4	0.269	−0.186	76.0
	Post	19.8			62.8
All workers	Pre	18.2	0.001	−0.317	73.7
	Post	22.3			56.1

CoVs showed that in post-treatment period, variations had been reduced in workers of all occupations in treatment group. The Cohen’s d ES showed moderate effect for all workers (d=−0.317). Similar ES observed for Rickshaw-pullers d=−0.281; Shop-keepers d=−0.501. Small ES was observed for Restaurant workers d=−0.-0.186).

Comparison between control and treatment group showed that WTP was significantly higher in treatment group in all occupations at 1% risk level. However, Independent sample t-test showed significant (10% risk-level) difference in mean WTP of rickshaw-pullers (Table 5).

**Table 5:** Difference in willingness-to-pay (mean and CoV) in control and treatment groups and within group effect sizes (Cohen’s d)

**Occupational group**	**Measurement**	**Mean**	**Sig. of mean difference (p-value)**	**ES (Cohen’s d)**	**Coefficient of variation**
Rickshaw-puller	Control	23.0	0.067	−0.293	69.2
	Treatment	27.2			45.2
Shop-keeper	Control	12.5	0.000	−0.606	112.9
	Treatment	20.3			57.1
Restaurant workers	Control	13.1	0.001	−0.537	96.3
	Treatment	19.8			62.8
All workers	Control	16.2	0.000	−0.433	92.8
	Treatment	22.3			56.1

CoVs were lower in treatment groups in all occupations compared with control groups. Moderate effect was found for all workers as well as for each occupational group.

#### Econometric analysis

Regression analysis (Table 6) shows that those who had gone through the educational intervention (treatment group) were willing to pay significantly more (33.8%) than workers in control group. Due to missing data in some control variables, number of observations reduced in the estimation. Using hadimvo test, 5 extreme outliers have been eliminated from the analysis. The significant difference between treatment and control group remain same even when the outliers were included. Workers who had up to primary level education are willing to pay less than those who had less than one year education. However, workers who had higher education than primary level are likely to pay more than reference group, but not significantly. A significant difference in WTP among occupational groups was observed. Restaurant workers and shop-keepers were willing to pay significantly less than rickshaw-pullers. No significant variation across geographic areas was found.

**Table 6:** Estimated effect of treatment (educational intervention) on willingness-to-pay (natural logged) for participating in health insurance

**Variables**	**Description**	**Coefficient (Std. Err.)**
Treatment	Yes (ref = control)	0.338 (0.064) ***
Age	In years	- 0.003 (0.004)
Gender	Female (Ref = male)	−0.221 (0.172)
Marital status	Unmarried (ref = married)	0.127 (0.085)
	Others (ref = married)	0.413 (0.385)
Household size	Number of household members	−0.018 (0.027)
Institutional educational level	Up to primary level (ref = less than one year)	−0.198 (0.083)**
	More than primary level (ref = less than one year)	0.056 (0.096)
Household income^a)^	Logged income per month	0.034 (0.043)
Illness in last 6 months	Illness of respondent or any household member	0.003 (0.099)
Location	Sub-district (ref= Metropolitan city)	0.119 (0.078)
	District (ref= Metropolitan city)	−0.122 (0.078)
Occupation	Shop worker (ref= Rickshaw-puller)	−0.387 (0.097)***
	Restaurant workers (ref= Rickshaw-puller)	−0.344 (0.084)***
Constant		3.402 (0.444)***
N		431
R-squared		0.156
F-value_(14,146)_ (Prob>F)		5.50 (0.000)
Mean VIF (max)		1.66 (2.68)
BP/Cook-Weisberg test (p>ch2)		10.31 (0.001)
Ramsey RESET, F (p>F)		0.82 (0.486)

Note: ***, ** and * denotes significant at 1%, 5% and 10% risk level respectively, ^a)^ Per equivalent adult (natural logged).

The regression model explains 15.6% of total variations (R^2^ = 0.156). The diagnostic tests favor the regression model. The Breusch–Pagan/Cook-Weisberg test showed that heteroscedasticity was not present in the model. Variance inflation factor (VIF) test with its maximum value of 2.68 indicates that there is no multicollinearity in the regression model. Ramsey RESET test showed that there was sufficient evidence against the hypothesis of omitted variable bias in the model.

For testing the robustness of the relationship between educational intervention and magnitude of WTP (natural logged), robust standard error has been calculated. The regression model has been reduced and extended by excluding and including variables. All models have shown that the workers in treatment group were willing to pay more for the health insurance that has been tested.

## Discussion

Among the workers in the treatment group, WTP increased between pre- and post-treatment periods. Both of these indicators were also higher in treatment groups in comparison to the control group. The WTP for participating in health insurance was 33.8% higher among workers who joined the educational intervention in comparison with who did not (control group). CoV of WTP was found to be generally lower in post-treatment period than in pre-treatment period. It was also lower in treatment group than in control group. Increase in WTP is significant, but low which reflects increase in the demand for insurance in a low-income community to some extent. However, the estimated effect size of this education intervention was found to be moderate in all occupational groups. In addition, lower CoV in treatment group means that the workers with educational intervention became more cohesive in their response to WTP, which can be considered as a positive outcome of the intervention program.

The workers who underwent the educational intervention are willing to pay on an average 5.48 BDT per week per household more than those who did not attend the intervention. It means that an additional 285 BDT (3.8 US$) can be accumulated per year from each worker. As an absolute amount, the mean WTP of a worker with educational intervention was estimated to be 21.7 BDT (0.30 US$) per week per household or 86.8 BDT (1.16 US$) per month, which was 16.2 BDT (0.22 US$) for workers in control group. In a one year period each worker with education on health insurance was willing to pay 1,128 BDT (15.2 US$). There are 41.5 million workers with informal employment (both urban and rural) in Bangladesh of which 20 million are in urban areas [9]. If all these workers can be brought into health insurance by educating them and the estimated WTP (premium) can be applied, a total sum of 22,568 million BDT (305 million US$) would be accumulated for financing their healthcare. The total health expenditure in Bangladesh is 191,486 million BDT (2,660 million US$). The estimated total amount of fund (320 million US$) from urban informal sector workers thus corresponds to 11.8% of total current healthcare expenditure of Bangladesh.

WTP for health insurance varies across countries. A study in Ghana shows that almost 64% of respondents were willing to pay about Cedi 5000 or 3.00 US$ per month for a household of five members for a National Health Insurance scheme aimed at the informal sector [19]. Asgary et al. (2004) examined WTP for health insurance in rural Iran finding that households are willing to pay on average 2.77 US$ per month for health insurance [34]. On average, an uninsured individual in the Greater Windhoek Area of Namibia is willing to pay 47.50 NAD or 6.60 US$ per capita per month [23]. A study in India showed that the median WTP was 600 INR per year (13.04 US$) per household or 1.09 US$ per month [20]. The studies above generally considered formal institutional education as an explanatory variable of WTP. But no educational intervention directed to “health insurance and usage of occupational solidarity” has been considered in earlier studies. It may not be surprising that a specific education on health insurance have higher impact than a formal education as found in our current study. We observed that the rickshaw-pullers are willing to pay more than other occupational groups. It may be explained by their daily cash-flow as income, while the other groups normally have a monthly-salary.

Our study has assessed the impact of health-insurance related educational intervention in terms of change/difference in WTP, while other studies simply did WTP for health insurance [19,20,23,34]. Since this current study mainly has assessed the impact of educational intervention in terms of change in WTP, it is not directly comparable to any earlier studies. Our literature review suggests that studies of such impact assessment are not found.

For impact assessment of education intervention quasi experimental study design is used. Quasi-experimental design is an empirical study which typically allows the researcher to control the assignment to the treatment condition, but using some criterion other than random assignment [35]. The major weakness of the quasi-experimental study design is the lack of random assignment. It requires multiple comparisons (between groups, within groups, effect sizes) while interpreting the results [36]. Due to non-randomness there is possibility of confounding bias in quasi-experimental which is often used to discount this study results. However, such bias can be controlled for using various statistical techniques such as multiple regression analysis [37]. Although the experimental design offer the greatest strength of evidence, quasi-experimental designs are often more feasible in natural social settings [35,38]. Utilizing quasi-experimental designs minimizes the threats to external validity as natural environments do not suffer the same problems of artificiality as compared to a well-controlled laboratory setting [38].

It can be argued that the control group might be contaminated by treatment group through discussing the intervention with each other. We, therefore, separated them by selecting from different geographic locations. Since shop-keepers and restaurant workers work in static physical facilities, they have low chance to be contaminated. Rickshaw-pullers in treatment and control groups were selected from garages (from where they hire rickshaw) located in different places. They may have small chance to meet each other in the street. Since they are mostly busy with their works during working hours, the risk of such contamination is low.

For encouraging workers to adopt health insurance, knowledge about health insurance is required. Workers need to understand that the health insurance is a way of financing healthcare through which healthcare can be availed at an affordable price whenever required. It reduces the catastrophic out-of-pocket health expenditure [39]. In a comprehensive educational module, a series of issues needs to be included. The target population should discuss their present need for healthcare and the ways of meeting healthcare expenses. The mechanisms (like, risk-pooling) of health insurance, the involvement and role of different actors in an insurance scheme (like, healthcare providers, insurance providers) and the nature of the prospective clients as well as the utility of insurance should be discussed in educational sessions. It is important to make clear distinguish between “knowledge about health insurance” and “marketing of a health insurance product” while educating the workers. Other relevant issues like trust to the insurance providers, benefit package, copayment/deductable should be discussed to a greater extent. It is also observed during educational sessions that most of disputation could be resolved through open discussion among workers. For more technical issues opinion of moderators of the sessions were useful. The workers in most of the cases could come into consensus.

## Conclusion

Educational intervention can be used for increasing demand for health insurance scheme using occupational solidarity among informal sector workers. Importantly, educational modules should be comprehensive covering need of healthcare in the community, existing accessibility to and quality of healthcare, risk-pooling mechanism, types of health insurance, strength of occupational solidarity, organization of health insurance using occupational cooperatives etc. The response to any query (does not matter how important the issue is) from the participating workers must be replied logically and convincingly during intervention sessions.

Healthcare financing is an essential component of universal health coverage. In low- and middle income countries funding healthcare for informal sector workers has appeared to be a challenge. Indirect taxes have emerged as a source of funding healthcare for these workers. The informal sector workers are not in the income-tax base though having income. Alternative funding sources are thus required. The government of Bangladesh and other low- and middle income countries can consider health insurance using occupational solidarity as a potential complementary source of funding along with indirect taxes for financing healthcare of informal sector workers.

## Competing interests

We have no competing interest.

## Authors’ contribution

JK contributed to conceptualizing the research idea and study design, literature search, data analysis, data interpretation, writing, revising and finalizing the manuscript. SA contributed to literature search, study design, data analysis, data interpretation, writing the manuscript. Both authors read and approved the final manuscript.
